# A Proteome Approach Reveals Differences between Fertile Women and Patients with Repeated Implantation Failure on Endometrial Level—Does hCG Render the Endometrium of RIF Patients?

**DOI:** 10.3390/ijms20020425

**Published:** 2019-01-19

**Authors:** Alexandra P. Bielfeld, Sarah Jean Pour, Gereon Poschmann, Kai Stühler, Jan-Steffen Krüssel, Dunja M. Baston-Büst

**Affiliations:** 1 Department of OB/GYN and REI (UniKiD), Medical Center University of Düsseldorf, Moorenstrasse 5, 40225 Düsseldorf, Germany; bielfeld@unikid.de (A.P.B.); sjboeddeker@gmx.de (S.J.P.); kruessel@unikid.de (J.-S.K.); 2 Molecular Proteomics Laboratory, Biomedical Research Centre (BMFZ), Heinrich-Heine-University, Universitätsstrasse 1, 40225 Düsseldorf, Germany; Gereon.Poschmann@uni-duesseldorf.de (G.P.); kai.stuehler@hhu.de (K.S.); 3 Institute for Molecular Medicine, University Hospital Düsseldorf, 40225 Düsseldorf, Germany

**Keywords:** endometrial receptivity, human chorionic gonadotropin, embryo transfer, window of implantation

## Abstract

Background: The molecular signature of endometrial receptivity still remains barely understood, especially when focused on the possible benefit of therapeutical interventions and implantation-related pathologies. Therefore, the protein composition of tissue and isolated primary cells (endometrial stromal cells, ESCs) from endometrial scratchings of ART (Assisted Reproductive Techniques) patients with repeated implantation failure (RIF) was compared to volunteers with proven fertility during the time of embryo implantation (LH + 7). Furthermore, an analysis of the endometrial tissue of fertile women infused with human chorionic gonadotropin (hCG) was conducted. Methods: Endometrial samples (*n* = 6 RIF, *n* = 10 fertile controls) were split into 3 pieces: 1/3 each was frozen in liquid nitrogen, 1/3 fixed in PFA and 1/3 cultured. Protein lysates prepared from fresh frozen tissue were processed for mass spectrometric analysis. Results: Three proteins (EPPK1, BCLAF1 and PTMA) showed a statistically altered abundance in the endometrial tissue of RIF patients. Furthermore, pathways like metabolism, immune system, ferroptosis and the endoplasmic reticulum were altered in RIF patients. Remarkably, endometrial tissues of RIF patients showed a significantly higher (*p*-value = 9 × 10^−8^) protein intensity correlation (Pearson’s correlation coefficient = 0.95) compared to fertile women (Pearson’s correlation coefficient = 0.88). The in vivo infusion of hCG stimulated proteins of endocytosis, HIF1 signalling and chemokine production. Notably, patients suffering from RIF had a clinical pregnancy rate of 19% after the intrauterine infusion of hCG before embryo transfer (ET) compared to their failed previous cycles. Conclusion: Our study showed for the first time that the endometrial proteome composition of RIF patients differs from fertile controls during the window of implantation. The intrauterine infusion of hCG prior to an embryo transfer might improve the chemokine triggered embryo-endometrial dialogue and intensify the angiogenesis and immune response. From a clinical point of view, the hCG infusion prior to an embryo transfer might increase the pregnancy rate of RIF patients.

## 1. Introduction

The molecular knowledge about a successful implantation of a human embryo into the receptive endometrium still remains unclear. Many studies tried to clarify the constitution and genomic expression of the receptive endometrium and embryonic development [1,2,3,4,5,6]. Taken together, it is all about timing and synchrony. In the clinical routine the question remains how to manage patients with repeated implantation failure (RIF) characterized with no anatomical pathologies, good response to treatment, good embryo quality but no occurrence of pregnancy [7,8]. The endometrial gene signature of RIF patients was described compared to patients that conceived during their first ART (Assisted Reproductive Techniques) cycle [9]. Significant changes occurred regarding cell division, chromosome segregation as well as cilium and signature genes. Endometrial and embryonic modifications or pathologies might cause RIF [8]. In order to improve the clinical outcome of ART patients several different strategies are applied in the daily clinical routine. Those approaches encompass the analysis and selection of embryos by identifying their euploidy via preimplantation genetic screening or improving embryo transfer (ET) techniques by applying an ultrasound guided method [10,11]. Another procedure focuses on influencing the endometrial receptivity. In this regard, methods like the endometrial injury in the cycle before the controlled ovarian stimulation to provoke an influx of immune competent cells [12] or the use of human chorionic gonadotropin (hCG) infusion prior to an embryo transfer are incorporated in therapy protocols but are discussed in contradictory terms in the literature [13,14]. HCG is produced by the syncytiotrophoblast and maintains the blood supply for the developing placenta. Moreover, the hyperglycosylated form is synthesized by the cytotrophoblast and plays a role as an autocrine factor regulating implantation, invasion and cellular growth [15]. Furthermore, hCG modulates the endometrial receptivity and the maternal immune system in vitro [13,14,15,16]. Therefore, we hypothesized that the endometrium of patients suffering from RIF might benefit from the infusion of hCG prior to ET. Hence, the aim of the study was to identify differences and similarities on protein level of the endometrium by performing a proteome approach between RIF patients and women with proven fertility (PF) as well as changes due to an hCG incubation in RIF patients. Moreover, the clinical outcome of the hCG infusion in RIF patients was investigated.

## 2. Results

### 2.1. Proteomic Analysis of Endometrial Samples and Decidualized Primary Endometrial Stromal Cells (dpESCs)

**Figure 1:**
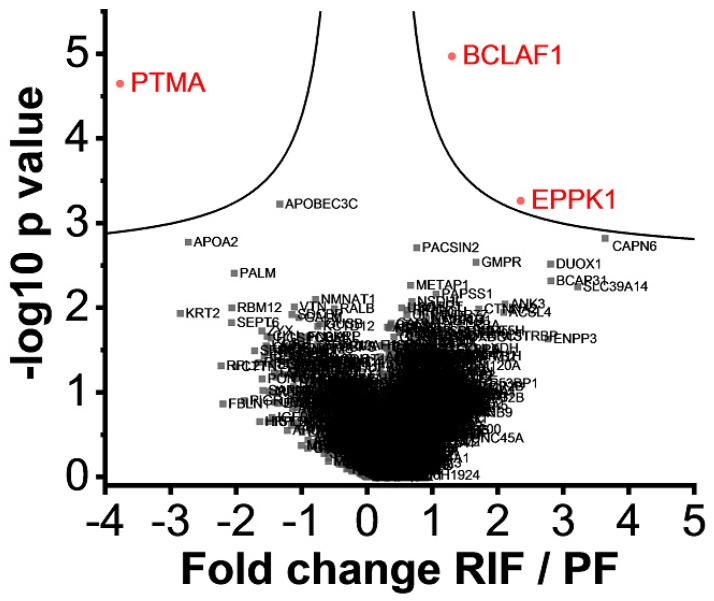
Volcano plots of the proteomic analysis of repeated implantation failure (RIF) endometrial tissue (*n* = 6) vs. proven fertility (PF) (*n* = 10). All identified proteins are shown with their associated gene names. Proteins showing a significantly altered abundance are highlighted in red.

**Figure 2:**
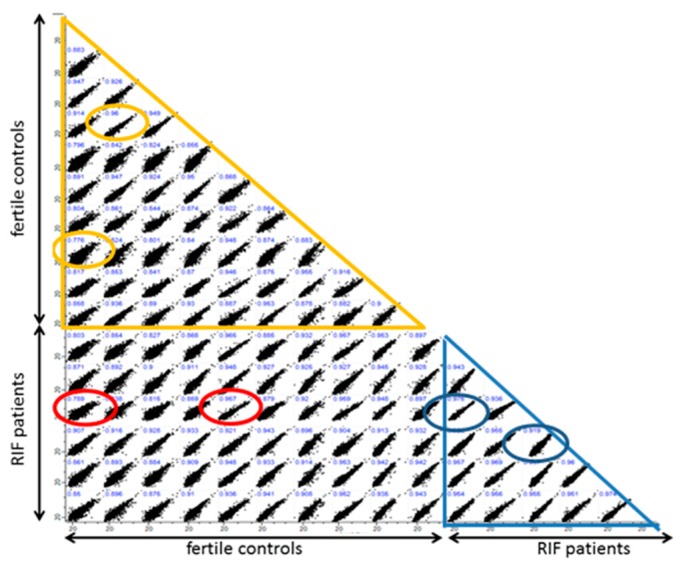
Intensity correlation (log 2 intensities are shown) of protein of endometrial tissue of RIF patients (*n* = 6) and PF (*n* = 10). Comparison within the RIF group is marked by a blue triangle and within the PF group by an orange triangle. Circles represent the highest and lowest correlation coefficient of the respective group comparison. The red circles highlight the highest and lowest correlation coefficient between RIF and PF.

**Figure 3:**
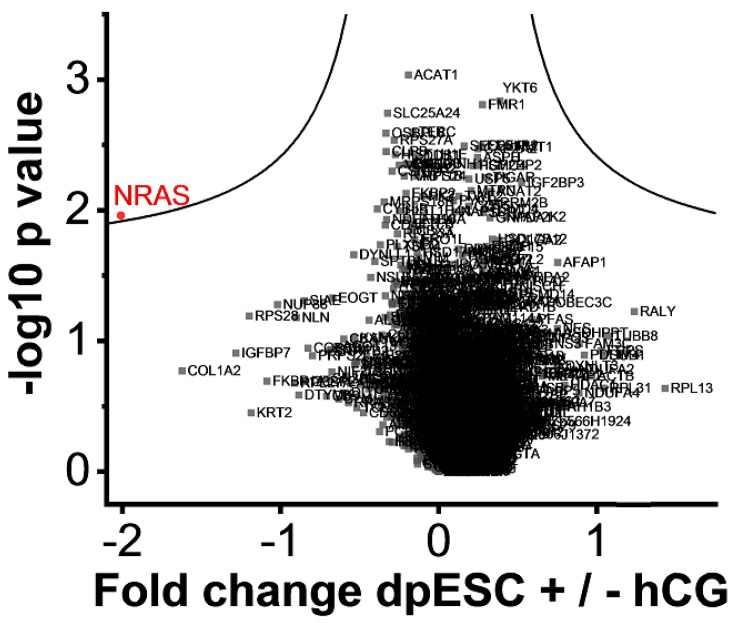
Volcano plots of cultured RIF decidualized primary endometrial stromal cell (dpESC) incubated for 24 h with and without 100 IU human chorionic gonadotropin (hCG)/mL. All protein names are shown, but only NRAS (marked in red) revealed a significant lower abundance after incubation with hCG.

**Figure 4:**
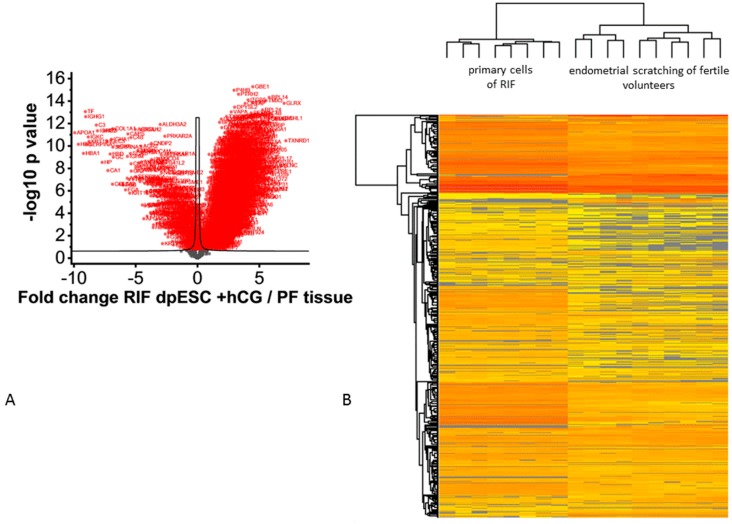
Volcano plot analysis of RIF dpESCs incubated with 100 IU/mL hCG vs. the endometrial tissue of PF (**A**). All proteins displayed in red show a statistically significant altered abundance. (**A**) heatmap (low to high abundance is coded by yellow to red color) representing the unsupervised hierarchical cluster analysis is shown in (**B**).

**Figure 5:**
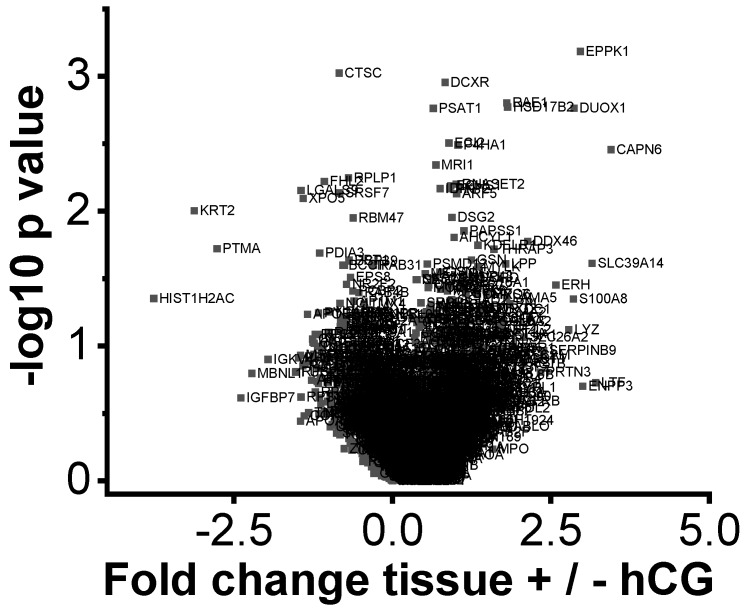
Results of the infusion with 500 IU hCG infusion for 24 h in PF.

Within this study, 4 different approaches were compared:

1. Proteomic analysis of endometrial biopsies of RIF patients (*n* = 6) vs. PF (*n* = 10). 2.120 proteins with sufficient quantitative data were identified in the first comparison between endometrial tissue from RIF patients and PF individuals (Figure 1). Three proteins could be identified with a statistically significant altered abundance in the endometrial tissue of RIF patients compared to PF by the significance analysis of the microarrFays method: a higher abundance of Epiplakin (EPPK1) and Bcl-2-associated transcription factor 1 (BCLAF1) and lower abundance of Prothymosin α (PTMA) (Figure 1). In addition, we found a significant (*p*-value = 9 × 10^−8^) variation of protein intensity correlation between the tissue samples of RIF and PF. Protein intensities from endometrial tissue of the secretory phase of RIF patients (*n* = 6) showed a mean Pearson’s correlation coefficient of 0.95 (range 0.92–0.97) whereas the tissue of PF showed a higher diversity (Pearson’s correlation coefficient = 0.88, range 0.78–0.96) (Figure 2).

2. dpESCs of RIF patients ± incubation with 100 IU hCG for 24 h (*n* = 8) to figure out if their protein signature is modifiable by hCG. Comparing RIF dpESCs incubated with 100 IU hCG/mL for 24 h to the initial samples, a statistically significant increase of the abundance of GTPase NRas (NRAS) could be observed (Figure 3).

3. dpESCs of RIF patients incubated with 100 IU hCG for 24 h (*n* = 8) vs. biopsies of PF (*n* = 10) to investigate a possible change towards the PF expression profile. Due to ethical reasons involving the use of RIF patient tissue in an ART treatment, we decided to retrieve the biopsies in a preceding cycle and culture the dpESCs in vitro. The analysis of the hCG-incubated RIF dpESCs (*n* = 8) vs. PF tissue samples (*n* = 10) led to 1.805 proteins showing a significantly different protein abundance between the 2 groups (Figure 4).

4. In vivo incubation with 500 IU hCG for 24 h vs. tissue of PF with proven fertility (*n* = 3) to determine hCGs’ effects in fertile endometrium. The sham procedure to mimic the hCG infusion on the day of the blastocyst transfer in vivo was conducted in 3 individuals of the PF group after measurement of the LH peak followed by the endometrial sampling 24 h after the hCG infusion. The endometrial protein expression was then further compared to their protein profiles obtained with the initial endometrial scratching (Figure 5). The in vivo incubation of hCG in PF resulted in no statistical differences (Figure 5).

### 2.2. Biosystemic Approach

Due to the small number of significantly different expressed proteins comparing the secretory phase endometrial tissues of RIF and PFs (Figure 1) and the in vivo incubation with hCG (Figure 5), we proceeded by analyzing the proteins showing a ± 1.3-fold altered abundance in the 4 different approaches in order to identify possible biosystems that were alike or varied between the groups (Table S1). Most of the 4 groups included proteins belonging to metabolism, immune system, cytoskeleton, ferroptosis, wound healing and apoptosis. Focusing on dpESCs of RIF and the effect of hCG, changes could be observed for cell cycle proteins, pathways and microRNAs in cancer, cytoplasm and cytokine signalling (Table S1).

### 2.3. Identification of Possible Key Players

Beyond the grouping according to biosystems we performed a literature search for the regulated proteins in the 4 groups that have been studied referring to key words (Table S2). Only a few proteins (ENPP3, IGFBP7, COL1A2) could be identified being regulated in more than one group (Table S2). ENPP3 was statistically significantly more abundant (2.78-fold) in RIF vs. PF tissue and in PF tissue after hCG incubation (3-fold) vs. the initial sampling (Table S2). Another significantly more abundant protein in RIF dpESCs and less abundant in PF tissue after hCG incubation was IGFBP7 (Table S2). COL1A2 could be identified as statistically significantly less abundant in RIF dpESCs. All other proteins presented in Table S2 were exclusively expressed in either tissues or dpESCs. The highest number of statistically significant different proteins was found in the comparison between RIF dpESCs + hCG and PF tissue (Table S2).

### 2.4. Clinical Data of Human Chorionic Gonadotropin (hCG) Infusion Prior to Embryo Transfer (ET)

To investigate the clinical effect of hCG on the endometrium, 16 RIF patients (mean age 39 years) treated with hCG infusion prior to ET between days 2 and 5 of embryo development which met the RIF criteria (>3 ETs with good quality embryos) were treated. In these 16 patients, the hCG infusion was performed 21 times (5 patients with more than 1 infusion). From these 16 RIF patients, 5 showed a positive hCG (>20 mU/mL) of which 4 hCG patients showed a clinical pregnancy including 1 abortion in the 7th week of pregnancy and 3 healthy singletons born.

## 3. Discussion

Unveiling the differences in the endometrial proteome signature between fertile women and patients suffering from RIF is a milestone from a clinical and scientific point of view. Identifying regulatory proteins will be helpful in counseling ART patients. Previous studies regarding the determination of the endometrial receptivity focused on gene or transcriptomic data and their validation for algorithms respectively [9,17,18,19,20]. Herein, we were able to identify EPPK1, BCLAF1 and PTMA, showing a statistically significantly altered abundance in endometrial tissue of PF and RIF. These proteins are neither included in the signature gene set tested of a dutch cohort nor in the proteome set tested in patients diagnosed as receptive or pre-receptive with the ERA^®^ test [2,9]. Therefore, we conclude that RIF seems to be an entity that is different from a pre-receptive state and which might not be identified with the ERA^®^ test, although an additional shifted individual timing remains contingent [21]. Another recent study confirmed our hypothesis that the entity RIF can not only be assumed to be a temporal problem, finding more genes with a reduced expression in the RIF group vs. controls [22]. In contrast to these gene data, we found a higher abundance of proteins in our RIF group pointing to the fact that gene and protein data are not necessarily congruent due to post-transcriptomic and post-translational regulation [23]. Beyond that, most of the cellular processes that vary between RIF and PF coincided when comparing the gene ontology data and our proteome data: cytoskeleton, cell cycle/cell survival [22]. Hence, similar processes were identified by others: cell adhesion, signalling, immune system, cytoskeleton and metabolism [9]. Furthermore, our proteome data cover cellular processes that were also determined in the ERA^®^ test like cytoskeleton, cell adhesion/cell junction, immune system, signalling and embryo implantation [24]. Regardless of the non-congruency between single genomic, transcriptomic and proteome data, the majority of biological and cellular processes that are known to be involved in endometrial receptivity can be detected and determined by different approaches. Regarding the question as to whether RIF patients benefit from an intrauterine hCG infusion prior to ET, RIF dpESCs were incubated with hCG. As expected, when the hCG-treated RIF dpESCs were compared to the PF samples, the mass spectrometry revealed a multitude of differently regulated proteins suggesting that hCG has an enormous influence on endometrial molecular composition. ENPP3 was one of the proteins showing a higher abundance in the tissue samples of RIF as well as in the tissue of PF after 24 h hCG incubation. ENPP3, which is higher abundant during the secretory phase and lower in endometriosis, is known to interact with nucleotides and modifies the glycosylation and, therefore, the activation state of other proteins [22,23,25]. ENPP3 itself can be triggered by hCG as shown by the in vivo incubation in the PF group and the higher abundance of ENPP3 in the tissue of RIF patients. Furthermore, it might hint at a generally higher activation state or rather a dysregulation in this entity. It was shown before that IGFBP7 is involved in the transformation of endometrial glands during the peri-implantation period and an inhibition of IGFBP7 in a mouse model led to lower implantation rates caused by the shift to an implantation-hostile Th1 immune response [24,26,27,28]. In our study, IGFBP7 was more abundant in RIF dpESCs incubated with hCG suggesting an implantation hostile response. By contrast, tissues of PF incubated with hCG in vivo displayed the anticipated physiologic downregulation of IGFBP7 triggered by the embryo signal. Proving the benefit of an hCG infusion in RIF patients, the examination of tissue samples of RIF after 24 h incubation with hCG would be preferable, because the higher abundance observed in dpESCs might be caused by cell isolation and culture. COL1A2 is the other protein that was less abundant in dpESCs of RIF incubated with hCG compared to no hCG. This downregulation is in accordance with a higher pregnancy chance in a cattle model and might, therefore, be a target of hCG in RIF tissue, too [29]. Concerning the protein intensity between RIF and PF tissue samples and the intra-individual expression pattern, this is the first study representing a significant variance in intensity correlation, a tighter expression pattern for RIF tissues and a wider range for PF samples. Regarding the clinical approach, we were able to show that when an austere classification of RIF patients was chosen for an intrauterine hCG infusion, the intervention led to a pregnancy rate of 19% per ET and an LBR of 14% per ET. This rise in pregnancy rate for those patients might be explained by an abolition of the endometrial asynchrony between the glands and stroma by delaying the premature stromal development caused by the controlled ovarian stimulation therapy [30]. It was shown before that an hCG-treated group showed no differences between the glandular and stromal staging when the endometrium was collected 5 days after oocyte retrieval, whereas the group without intervention showed a 2-day asynchrony in glandular vs. stromal development [31]. This group additionally connected the histological findings to upregulated mRNA levels of the steroid receptors ESR1 and PGR in the hCG-treated group. They further showed an upregulation of α-SMA protein in the sub-luminal epithelial and the peri-vascular stromal cells where its expression prevents stromal apoptosis and, rather, regulates its differentiation during decidualization. ACTIN seems to be a target of cell reorganization during decidualization and pre-implantation period as we saw the upregulation of ACTIN in dpESCs of RIF patients after incubation with hCG [32]. Since a proper decidualization is one of the crucial key processes for the embryo implantation, hCGs’ function herein could, therefore, be hypothesized. The results regarding hCGs effectiveness in enhancing ART results are still conflicting [33]. The inconclusiveness is attributed to the heterogeneity of the trials (dosage, timing, origin of hCG source (urinary vs. recombinant) and developmental stage of embryos transferred). A sub-group investigation of the current Cochrane database analysis supports a benefit of a minimum of 500 IU hCG infused before cleavage stage embryos are transferred [13]. However, all babies born in our clinical setting resulted from blastocyst transfers and infusion of 500 IU hCG 10–15 min before transfer. Therefore, a well-powered randomized controlled trial with either a comparable time frame for the hCG infusion before cleavage stage and/or blastocyst ET or repeated hCG infusions before blastocyst ET should be performed to answer this question.

## 4. Materials and Methods

### 4.1. Identification of Repeated Implantation Failure (RIF) and Proven Fertility (PF) Individuals

Endometrial tissues were obtained from RIF patients undergoing infertility treatment and from PF via endometrial scratch biopsies on days 19–23 of the menstrual cycle (LH + 7 according to their cycle length, ultrasound guided cycle determination assessed by 2 independent physicians). 10 PF individuals (age 35–43 years) were recruited with a history of at least 1 (up to 3) live birth after spontaneous conception. Six RIF patients were included with at least 3 unsuccessful ETs with good quality embryos (age 32–43 years). Exclusion criteria were irregular menses, hormonal/mechanical contraception within 3 months prior to endometrial biopsy, polycystic ovary syndrome and endometriosis. The study was conducted in accordance with the Declaration of Helsinki and has been approved by the Ethics Committee of the Heinrich-Heine University Düsseldorf (5528R [2016-06-16], 4394R [2016-05-24]) and all individuals gave written consent before participating.

### 4.2. Extraction of Human Primary Endometrial Stromal Cells (pESCs)

Endometrial samples were collected into DMEM-F12 with 15 mM HEPES, supplemented with 1% penicillin/streptomycin and 1% amphotericin b (all Biowest, Nuaille, France), rinsed with HBSS and split into 3 pieces. 1/3 of the sample was frozen in liquid nitrogen for later mass spectrometric analysis, 1/3 fixed in 4% paraformaldehyde (Carl Roth GmbH & Co KG, Karlsruhe, Germany) and 1/3 used for primary endometrial stromal cells (pESC). The sample was cut into <1 mm pieces, digested with 1 U/mL collagenase IV (Sigma-Aldrich/Merck, Darmstadt, Germany) for 1 h at 37 °C. The suspension was passed through a 40 μm sieve and the flow through pelleted at 300× *g* for 5 min. Cells were resuspended and transferred to a 35 mm culture dish (Sarstedt AG & Co., Nümbrecht, Germany). PESCs grew more than 5 days before subculturing and passages 3 to 6 have been used.

### 4.3. Cell Culture Conditions

Cells were maintained at 5% CO_2_ and 37 °C and 20% O_2_, whereas experiments were perfomed with low oxygen conditions (4%). PESCs were cultured in DMEM-F12 with 15 mM HEPES and stable glutamine, supplemented with 10% (*v*/*v*) FCS, 1% (*v*/*v*) penicillin/streptomycin, 200 μM sodium pyruvate, 50 μg/mL gentamicin sulfate (all Biowest, Nuaille, France) and 20 μg/mL insulin (Sigma-Aldrich). PESCs were decidualized with 0.5mM 8-bromo-cAMP (Biolog, Bremen, Germany) and 1 μM medroxyprogesterone 17-acetate (MPA; Sigma-Aldrich) for 6 days. Decidualization was proven morphologically via bright field microscope (Leica, Wetzlar, Germany) and biochemically via detection of prolactin running the Human Prolactin DuoSet enzyme-linked immunosorbent assay (ELISA, R&D Systems, Abingdon, UK) according to the manufacturer’s protocol. Supernatants from successful decidualized samples ranged from 0.7 to 4.4 ng/mL.

### 4.4. Protein Analysis of pESCs

PESCs were decidualized, followed by incubation with 100 U/mL hCG (Sigma-Aldrich) for 24 h, harvested with 2% (*v*/*v*) trypsin/EDTA and washed with phosphate-buffered saline (PBS) and stored at –80 °C until preparation for mass spectrometric analysis.

### 4.5. Mass Spectrometric Protein Identification and Quantification

Protein lysates from endometrium tissue and cell samples were prepared and processed as described [34]. Briefly, 5 μg protein lysates were separated over a 5 mm running distance in a polyacrylamide gel and, after silver staining, protein containing bands were processed including reduction and alkylation with iodoacetamide and tryptic digestion into peptides. 

Peptides were finally resuspended in 0.1% trifluoroaceticacid and 500 ng peptides separated by nano liquid chromatography on an Ultimate 3000 rapid separation liquid chromatography system (Thermo Scientific, Dreieich, Germany) using a h gradient essentially as described [35]. The liquid chromatography system was online coupled with an Orbitrap Elite mass spectrometer (Thermo Scientific) equipped with a nano electrospray source. The mass spectrometer was operated in data-dependent positive mode, with a source voltage of 1.4 kV and capillary temperature of 275 °C.

Survey scans were recorded in the Orbitrap at a resolution of 60,000 (at 400 *m*/*z*) and per cycle the 20 top intense 2- and 3-fold charged precursor ions were isolated, fragmented by collision induced dissociation and analyzed in the linear ion trap part of the instrument with a resolution of 5400 (at 400 *m*/*z*).

Mass spectrometric data was processed within the MaxQuant environment (version 1.5.7.0, MPI for Biochemistry, Planegg, Germany) using standard parameters if not stated otherwise. Searches were carried out in the UP000005640 human proteome dataset downloaded from the UniprotKB on 3 February 2017 considering tryptic cleavage specificity as well as carbamidomethyl on cysteines as fixed and methionine and N-terminal acetylation as variable modifications. Proteins and peptides were accepted at a false discovery rate of 1%, and only proteins considered which have been identified with at least two peptides. Precursor-based quantification was enabled by the label-free quantification algorithm implemented in MaxQuant using the ‘match between runs’ feature.

### 4.6. hCG Infusion Prior to an ET in RIF Patients and hCG In Vivo Infusion in PF

The infusion of 500 IU urinary hCG (resolved in the corresponding diluent; Brevactid^®^, Ferring GmbH, Kiel, Germany) was conducted 15 min before the ET. The first endpoint was the biochemical measurement of hCG. The second endpoints were ongoing pregnancy rate and documented live birth. HCG infusion in PF (500 IU as above) was performed 7 days after LH peak representing the window of implantation. Sampling was conducted 24 h later and the tissue was snap-frozen for proteome analysis.

### 4.7. Statistical Analyses

Log 2 transformed normalized intensities from the label-free quantification were further analyzed within the Perseus software package (version 1.5.8.5, MPI for Biochemistry). Besides supervised and unsupervised hierarchical cluster analysis, comparisons of different groups were carried out. Here, only proteins were considered showing in at least one group 4, 7, 6, 7 valid values respectively for comparisons 1–4. Missing values were imputed using a downshifted (1.8 standard deviations) normal distribution (width 0.3 standard deviations) and Student’s *t*-test calculated in combination with the significance analysis of microarrays method implemented in Perseus using an S_0_ of 0.1 and false discovery rate of 5% [36]. This method considers both the *p*-value and fold-change for the determination of statistical significance and accounts also for false positives by multiple testing using a permutation based strategy for the determination of the false discovery rate. 

Proteins differing in their abundance between the groups were further descriptively analyzed for biosystemic involvement (National Center for Biotechnology Information (NCBI) biosystems database; https://www.ncbi.nlm.nih.gov/biosystems/) and already published functions with regard to endometrium, endometriosis, decidua, pregnancy and embryo using a pubmed literature search (https://www.ncbi.nlm.nih.gov/pubmed).

## 5. Conclusions

For the first time, we showed that the endometrial proteome composition of RIF patients differs from fertile controls during the window of implantation. The intrauterine infusion of hCG prior to embryo transfer might improve the chemokine triggered embryo–endometrial dialogue. From the clinical point of view, the hCG infusion prior to embryo transfer might increase the pregnancy rate of RIF patients.

## Supplementary Materials

Supplementary materials can be found at http://www.mdpi.com/1422-0067/20/2/425/s1.
