# Supplementary material for: A Proteome Approach Reveals Differences between Fertile Women and Patients with Repeated Implantation Failure on Endometrial Level—Does hCG Render the Endometrium of RIF Patients?
Source: Int J Mol Sci. 2019 Jan 19;20(2):425. doi: 10.3390/ijms20020425 (PMC6358950; doi:10.3390/ijms20020425)
Supplement: Supplementary file 1 [file ijms-20-00425-s001.pdf]

Supplementary Material

Table S1: Biosystems with proteins showing an altered abundance in endometrial tissue (+/- hCG) and/or ESCs (+/- hCG).

biosystem	tissue of RIF vs. tissue of fertile controls	cells of RIF +/-hCG	cells of RIF +hCG versus tissue of fertile controls	tissue of in vivo hCG-incubation vs. tissue of fertile controls
metabolism	•	•	•	•
immune system	•	•	•	•
cytoskeleton	•	•	•	•
endoplasic reticulum	•			
ferroptosis	•		•	•
wound healing	•	•		•
response to stimulus	•		•	
apoptosis/cell death	•		•	•
cell adhesion/cell junction	•		•	
proteoglycans	•		•	
biological regulation		•		
gene expression		•		
cell cycle		•	•	
pathways/microRNAs in cancer		•	•	
cytoplasm		•	•	
developmental biology		•		
cytokine signaling		•	•	
secretion			•	
estrogen signaling	•			
TNFα/NfkappaB signaling	•			
PPAR signaling	•			
embryo implantation	•			
intracellular transport		•		
respiratory chain		•		
signal transduction		•		
cell growth		•		
transmembrane transport		•		

membrane			•	
extracellular transport			•	
response to stress			•	
deubiquitination			•	
homeostasis			•	
glycolysis			•	
insulin signaling			•	
misregulation in cancer			•	
endocytosis				•
HIF1 signaling				•
chemokine production				•

Table S2: Proteins that have been published and were associated with following categories: endometrium, decidua, placenta, embryo and/or endometriosis (arrows ↓lower abundant in the comparison, ↑ higher abundant; * statistical significance $p < 0.05$).

proteins published in endometrium, decidua<br><br>pregnancy, placenta, embryo, endometriosis	tissue of RIF vs. tissue of fertile controls	cells of RIF +/-hCG	cells of RIF +hCG versus tissue of fertile controls	tissue of *in vivo* hCG-incubation vs. tissue of fertile controls
ENPP3	↑*			↑*
EPPK1	↑*			
BCLAF1	↑*			
HLA-A	↑			
FKBP5	↑			
PTMA	↓*			
APOA2	↓			
Fibulin-1	↓			
PIGR	↓			
VTN	↓			
PON1	↓			
PAM	↓			
IGFBP7		↑	↑*	↓
RPS28		↑		
NUP88		↑		
SIAE		↑		
ALCAM		↑		
NSUN2		↑		
GOLGA2		↑		
HIST1H4A		↑		
PHB2		↑		
RPS27A		↑		
OSBPL8		↑		
TFRC		↑		
SLC25A24		↑		
ACAT1		↑		
NRAS		↓*		
COL1A2		↓	↓*	
RPL13		↓		
TUBB8		↓		
NDUFA4		↓		
HDAC1		↓		
NDUFA2		↓		
NES		↓		
AASDHPPT		↓		
HSD17B12		↓		
FMR1		↓		
TIGAR		↓		
UCHL1			↑*	
GLRX3			↑*	
TXNRD1			↑*	
CD 59			↑*	
AKR1B1			↑*	
NNMT			↑*	
PBXIP1			↑*	
HK2			↑*	
MMP3			↑*	
NQO1			↑*	
Actin			↑*	
ITGB5			↑*	
CUL4B			↑*	

RPS6			↑*	
LEPRE1			↑*	
TAGLN			↑*	
Serotransferrin			↓*	
IGHG1			↓*	
IGHA1			↓*	
HBD			↓*	
CKB			↓*	
Serpin A3			↓*	
CAPS			↓*	
ORM1			↓*	
CAT			↓*	
GSN			↓*	
ACOX1			↓*	
APOA4			↓*	
SNX5			↓*	
KRT18			↓*	
OGN			↓*	
EPCAM			↓*	
HIST1H2BN			↓*	
DSP			↓*	
ACSL5			↓*	
C3			↓*	
SERPIN G1			↓*	
Serpin A1			↓*	
APOA1			↓*	
HPX			↓*	
CA2			↓*	
ITIH4			↓*	
ITIH1			↓*	
ADD1			↓*	
PPCD4			↓*	
GC			↓*	
CA1			↓*	
TTR			↓*	
FGG			↓*	
FGA			↓*	
ALDH1A2			↓*	
PRKAR2A			↓*	
ASRGL1			↓*	
SORD			↓*	
GUSB			↓*	
C4BPA			↓*	
LTF				↑*
MYLK				↑
MAOA				↑
PTMA				↓
Galectin-9				↓
XPO5				↓
CTSC				↓
PDIA3				↓
